# Evaluation of Macroalgae Sulfated Polysaccharides on the *Leishmania (L.) amazonensis* Promastigote

**DOI:** 10.3390/md11030934

**Published:** 2013-03-20

**Authors:** Camila Lehnhardt Pires, Selma Dzimidas Rodrigues, Daniel Bristot, Henrique Hessel Gaeta, Daniela de Oliveira Toyama, Wladimir Ronald Lobo Farias, Marcos Hikari Toyama

**Affiliations:** 1 São Vicente Unit, State University of São Paulo Julio Mesquita Filho, UNESP, Praça Infante Dom Henrique, s/n, São Vicente 11330-900, SP, Brazil; E-Mails: sdr1@clp.unesp.br (S.D.R.); dbristot@hotmail.com (D.B.); henriquehg@gmail.com (H.H.G.); mhtjpn@gmail.com (M.H.T.); 2 Center of Biological and Health Sciences, Mackenzie Presbyterian University, Rua da Consolação, 896, São Paulo 01302-907, SP, Brazil; E-Mail: gaveira@yahoo.com.br; 3 Pici Unit, Federal University of Ceará, UFC, Av. Mister Hull, s/n, Bloco 827, Fortaleza, CE, 60356-000, Brazil; E-Mail: wladimir@ufc.br

**Keywords:** macroalgae, *Leishmania (L.) amazonensis*, sulfated polysaccharides, *Solieria filiformis*, *Botryocladia occidentalis*, *Caulerpa racemosa*, *Gracilaria caudate*

## Abstract

The sulfated polysaccharides from *Solieria filiformis* (Sf), *Botryocladia occidentalis* (Bo), *Caulerpa racemosa* (Cr) and *Gracilaria caudata* (Gc) were extracted and extensively purified. These compounds were then subjected to *in vitro* assays to evaluate the inhibition of these polysaccharides on the growth of *Leishmania (L.) amazonensis* promastigotes. Under the same assay conditions, only three of the four sulfated polysaccharides were active against *L. amazonensis*, and the polysaccharide purified from Cr was the most potent (EC_50_ value: 34.5 μg/mL). The polysaccharides derived from Bo and Sf demonstrated moderate anti-leishmanial activity (EC_50_ values of 63.7 μg/mL and 137.4 μg/mL). In addition, we also performed *in vitro* cytotoxic assays toward peritoneal macrophages and J774 macrophages. For the *in vitro* cytotoxicity assay employing J774 cells, all of the sulfated polysaccharides decreased cell survival, with CC_50_ values of 27.3 μg/mL, 49.3 μg/mL, 73.2 μg/mL, and 99.8 μg/mL for Bo, Cr, Gc, and Sf, respectively. However, none of the sulfated polysaccharides reduced the cell growth rate of the peritoneal macrophages. These results suggest that macroalgae contain compounds with various chemical properties that can control specific pathogens. According to our results, the assayed sulfated polysaccharides were able to modulate the growth rate and cell survival of *Leishmania (L.) amazonensis* promastigotes in *in vitro* assays, and these effects involved the interaction of the sulfated polysaccharides on the cell membrane of the parasites.

## 1. Introduction

The surface of protozoan parasites, such as those belonging to the parasite genus *Leishmania*, is responsible for regulating interactions with the extracellular environment and is involved in the absorption of nutrients and signaling pathways. According to Azevedo-Pereira *et al*. [1], there is evidence that heparin-binding proteins (HBPs) present on the surface of *Leishmania* spp*.* may play important roles in the life cycle of parasites and in defining the success of parasite attachment to and invasion of tissues of the mammalian and invertebrate hosts. In addition, there is evidence that HBPs can promote internalization and signaling in the host cells during the infection time course. More recently, de Castro Côrtes *et al*. [2] isolated metallo-protease protein fractions from the flagella or membrane promastigotes of *L. (V) braziliensis* that were capable of forming stable complexes with glycosaminoglycans (heparin-like activity). Additionally, de Castro Côrtes *et al*. [3] showed that these HBPs modulate the activity of signaling in the cellular environment and play specific roles in the host-parasite interactions of *Leishmania (V.) braziliensis*.

Although heparin is not found on the surface of host cells, this glycosaminoglycan (GAG) has been commonly used as a tool for studying pathogen-host cell interactions. It was previously demonstrated that amastigotes of *L. (L.) amazonensis* and *Leishmanai (L.) major* have a greater ability to bind to heparin than promastigotes of these same species. In addition, GAGs, including heparin, can induce the proliferation of *L. (L.) major* in the gut of the insect vector, increasing the load of experimentally infected insects. There is evidence that the HBPs present on the surface of *Leishmania* spp. can play an important role in the life cycle of parasites, defining the success of parasite binding and invasion of the host tissues of mammals and invertebrates. In the parasite species in which these proteins were identified, the HBPs act as adhesion proteins and can promote internalization and signaling in host cells [2].

Marine macroalgae have high concentrations of sulfated polysaccharides (SP), heterogeneous and complex macromolecules with significant importance to the physiology of algae. These compounds have been explored in the biomedical field, indicating the therapeutic potential of sulfated polysaccharides [4]. In this respect, extracts produced from green, brown, and red algae have already demonstrated significant activity against fungi, bacteria, viruses and protozoa [5,6]. However, until now, few works have demonstrated the anti-leishmanial activity of seaweed extracts. Recently, results have shown that the selective index of extracts is very high in different experimental models, suggesting low toxicity of algae extracts to mammalian cells, which in turn can be explored to develop new candidate drugs against diseases and to examine different pathological conditions. In this study, we evaluated the effects of four highly purified sulfated polysaccharides from *Solieria filiformis* (Sf), *Botryocladia occidentalis* (Bo), *Caulerpa racemosa* (Cr) and *Gracilaria caudate* (Gc) on the growth of *Leishmania (L.) amazonensis* promastigote, as well as their cytotoxic effects.

## 2. Results and Discussion

**Figure 1:**
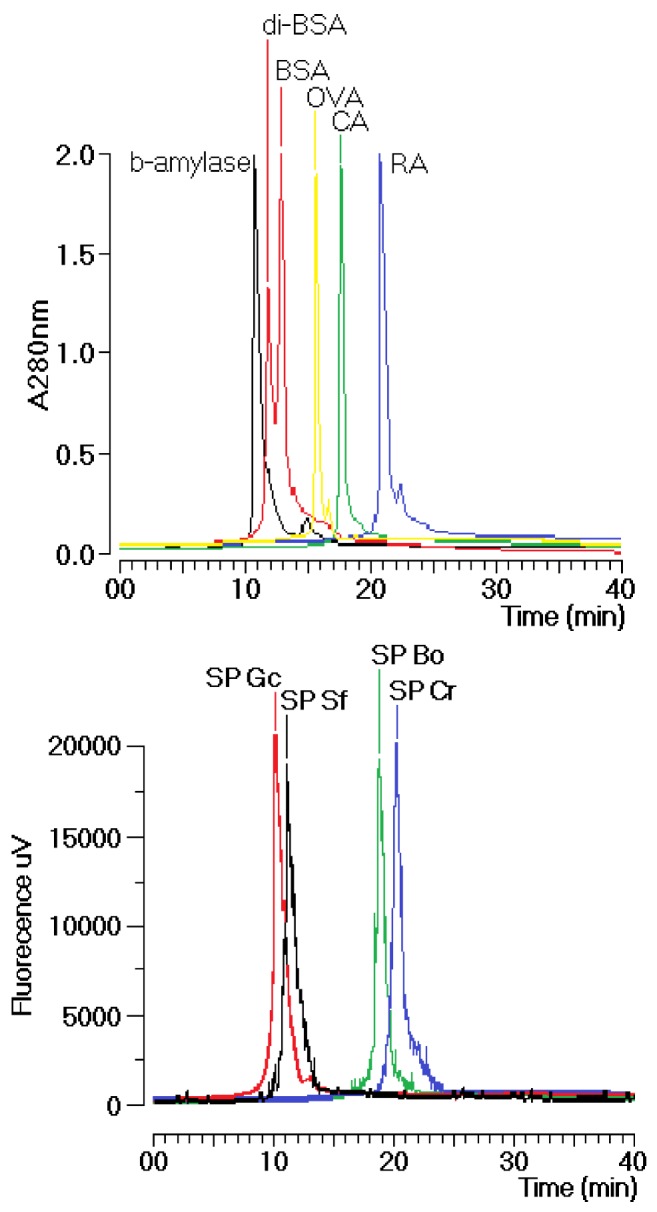
Chromatographic profile of the size exclusion fractionation of sulfated polysaccharides in TSKgel G3000SW silica-based GFC columns. Samples of 1 mg/mL were dissolved in phosphate buffer and centrifuged at 4500× *g* for 5 min, and the supernatant was applied to the column. The flow rate was maintained at 1 mL/min, and the eluent was monitored by fluorescence. The purified fractions of the sulfated polysaccharides were named SP.

The sulfated polysaccharides used in this study were extensively purified from the crude extracts of *Solieria filiformis* (Sf), *Botryocladia occidentalis* (Bo), *Caulerpa racemosa* (Cr) and *Gracilaria caudate* (Gc) by Diethylamionoethyl (DEAE) ion exchange chromatography. These fractions were then subjected to a new chromatography process using a TSKgel G3000SW column with a molecular size exclusion stationary phase previously equilibrated with phosphate buffer. The size exclusion chromatographic analyses of the sulfated polysaccharides (SP) fractions from the DEAE column of Gc, Sf, Bo and Cr showed several fractions, including one main peak that corresponded to approximately 35% of the whole fraction (Figure 1). The molecular mass of Gc, Sf, Bo and Cr were estimated to be greater than 200 kDa, 30 kDa and 25 kDa, respectively, according to the molecular mass markers: blue dextran (2000 kDa); β-amylase (225 kDa); and bovine serum albumin (BSA, Sigma, 66 kDa), which forms a natural dimer of 120 kDa; ovalbumin (OVA, 43 kDa); carbonic anhydrase (CA, 29 kDa); and ribonuclease (RA, 14 kDa). 

**Figure 2:**
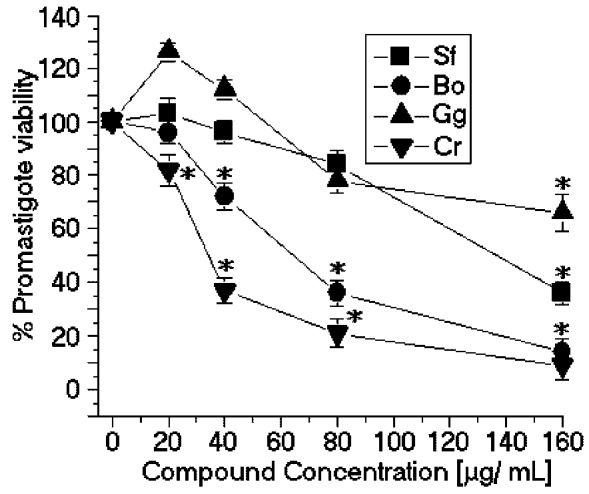
Anti-promastigote effects of SP from the seaweeds *Solieria filiformis* (Sf), *Botryocladia occidentalis* (Bo), *Caulerpa racemosa* (Cr), and *Gracilaria caudata* (Gc) on *L. (L.) amazonensis*. * *p* < 0.05 indicates significant anti-promastigote effects compared to untreated promastigote forms.

**Figure 3:**
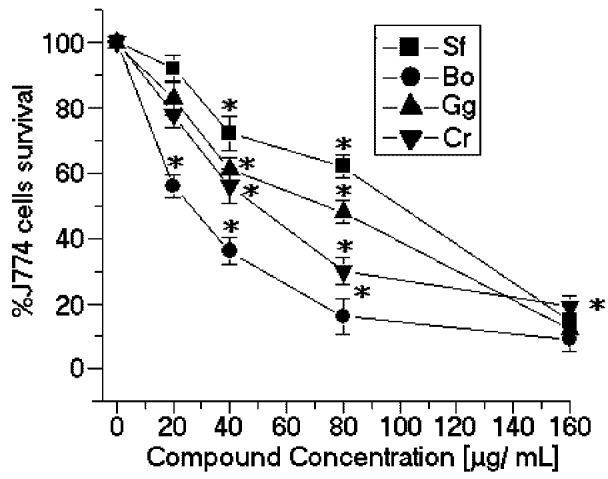
Cytotoxic effects of SP from the seaweeds Sf, Bo, Cr, and Gc on J774 macrophages. * *p* < 0.05 indicates significant cytotoxic effects compared to untreated J774 cells.

The SPCr significantly reduced the viability of the promastigotes of *L. (L.) amazonensis* in a dose-dependent manner and showed a low EC_50_ (34.5 μg/mL) (Figure 2). Previous studies showed that the crude extracts of cold water *Caulerpa sertularioides* inhibited the growth rate of the promastigotes of *Leishmania major* and had an EC_50_ of 65 μg/mL [7] (Table 1). Moreover, the methanol extract of *Caulerpa racemosa* increased the leishmanicidal effect on the promastigotes of *L. donovani* and was able to eliminate 50% of the promastigotes with a dose of 22.6 μg/mL of extract [8]. Additionally, the SP *C. racemosa* used in this study showed moderate cytotoxic effects in the J774 macrophage cell line (CC_50_ = 49.3 μg/mL) (Figure 3). These results suggest that SPCr may present potential leishmanicidal activity. These results can be extended by the studies of Süzgeç-Selçuk [8], which have shown that concentrations higher than 90 μg/mL of a crude extract of methanol were required to remove 50% of the L6 cell population. The chemical heterogeneity of this fraction found in the crude extract and the sensibility of cell lineage should result in a higher EC_50_ than those found in the present work.

SPBo also significantly reduced the viability of the promastigotes of *L. (L.) amazonensis* in a dose-dependent relation and showed a lower EC_50_ (58.8 μg/mL) (Figure 2). Although these results demonstrated that the sulfated polysaccharides showed good activity against the growth of *L. (L.) amazonensis*, there are few studies showing other applications of algae of the genus *Botryocladia*. There are some studies with *B. leptopoda*. One of these studies showed that the crude extract and hexane fraction brought about a marked reduction in the peripheral microfilarial level in the rodent filarial parasites [9]. Moreover, studies with steroidal alkaloids from *B. leptopoda* showed moderate *in vitro* anti-leishmanial activity of approximately 60.81 ± 0.41 μg/mL [10]. Recent studies by Sabina *et al*. [11] showed that ethanol extracts of algae, including *B. leptopoda*, also showed significant *in vitro* anti-leishmanial activity. The analysis of the results obtained from the *in vitro* treatment of *L. (L.) amazonensis* promastigotes with SPBo were significantly better than the *in vitro* anti-leishmanial activity from secondary metabolites because the molecular weights of the natural compounds are smaller than 1 kDa and the sulfated polysaccharides of *B. occidentalis* had a molecular mass of 30 kDa. Studies conducted by Sabina *et al*. [11] identified that the flavone extracted from the red seaweed *Osmundea pinnatifida* showed inhibition of viability in tests with *Leishmania*. The SP from *B. occidentalis* had the second most potent cytotoxic effects on the J774 macrophage cell line (CC_50_ = 27.3 μg/mL) (Figure 3) and the lowest selectivity index (SI = 0.42) (Table 1). 

**Table 1:** Determination of the EC_50_ and CC_50_ values and selectivity indices of the purified sulfated polysaccharides in comparison with Amphotericin B.

Algae species	EC50 (μg/mL)	CC50 (μg/mL)	Selectivity index
*C. racemosa*	34.5	49.3	1.42
*B. occidentalis*	63.7	27.3	0.42
*S. filiformis*	137.4	99.8	0.72
*G. caudate*	No activity	73.2	Not determined
Amphotericin B	0.05	≥90	-

In contrast, the SP from *Solieria filiformis* presented the highest anti-leishmanial values (EC_50_ = 137.4 μg/mL) (Figure 2) and cytotoxic activities (CC_50_ = 99.8 μg/mL) (Figure 3). The selectivity index was 0.72 μg/mL, thus suggesting that the cytotoxicity is responsible for killing the leishmania, rather than being a direct effect on the promastigote forms (Table 1). The SP from *Gracilaria caudata* algae showed no anti-leishmanial effect; however, a CC_50_ of 73.2 μg/mL was reached. Studies by Fouladvand *et al*. [7] investigated the IC_50 _values for the hot water extracts of *Gracilaria corticata* (IC_50_ ≤ 38 μg/ mL), *Gracilaria salicornia* (IC_50_ ≤ 46 μg/ mL) and *Gracilaria salicornia* (IC_50_ > 105 μg/mL) and the cold water extract of *Gracilaria corticata* (IC_50_ > 74 μg/mL). Studies on the anti-leishmanial effects of Mediterranean red algae showed that halogenated compounds from these algae have strong leishmanicidal activities [7].

**Figure 4:**
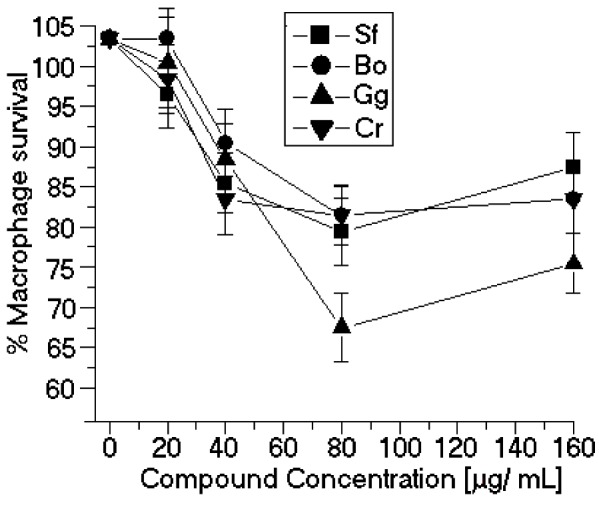
Cytotoxic effects of SP from the seaweeds Sf, Bo, Cr, and Gc on peritoneal macrophages. * *p* < 0.05 indicates significant cytotoxic effects compared to untreated peritoneal macrophage.

The seaweed-derived SP tested in this study significantly inhibited the promastigote growth rate *in vitro*. Some of the observed IC_50_ values were comparable to those of other natural products, such as other organic small molecules isolated from algae sources. This article is the first report of the anti-leishmanial activity of algae SP. Our results also showed that none of the SP assayed here significantly modified the peritoneal macrophage viability (Figure 4). The difference between the cytotoxicity of the J774 macrophages and macrophages collected from the peritoneum is the resulting heterogeneity in the intracellular survival among the various macrophage-sensitive mutants, which may reflect the relative importance of the individual mutated genes in survival within macrophages [12]. The *in vitro* anti-leishmanial activity of sulfated polysaccharides depends on the molecular size of the sulfated polysaccharides. Our results show that the SP with high molecular weights showed the worst inhibition results. In the case of the sulfated polysaccharides of *Botryocladia occidentalis* and *Caulerpa racemosa*, the difference in molecular weight is small; therefore, the chemical composition of both sulfated polysaccharides can determine the best *in vitro* anti-leishmanial activity.

## 3. Experimental Section

### 3.1. Specimen Collection

*Solieria filiformis* (Sf), *Botryocladia occidentalis* (Bo), *Caulerpa racemosa* (Cr) and *Gracilaria caudata* (Gc) were collected from the coast of Fortaleza, Brazil during the summer. After collection, all samples were washed thoroughly under tap water, packed in plastic bags, transported to the laboratory on dry ice, washed with Milli Q water, and stored at −80 °C until the extracts were prepared. The fraction of SP from seaweed was supplied by Prof. Dr. Wladimir R. L. Farias (group of Department de Engenharia de Pesca/Biologia Molecular e Biologia Marinha, UFC, Fortaleza, Ceará, Brazil). 

### 3.2. Fractionation of the SP from Algae

All of the dry seaweed (5 g) was triturated and hydrated in 250 mL of 0.1 M sodium acetate, 5 mM cysteine and 5 mM EDTA, pH 5.0. Immediately afterward, 17 mL of crude papain solution (30 mg/mL) was added, and the mixture was incubated in a water bath at 60 °C for 24 h. The material was then filtered and centrifuged (14,000 rpm, 30 min, 4 °C). After this step, 16 mL of cetylpiridinium chloride (CPC) was added to provide a final concentration of 10%. The solution was incubated at room temperature for 24 h to allow the polysaccharides to precipitate. The SPs were washed with 500 mL CPC, dissolved in 174 mL of 2 M NaCl:ethanol (100:15, v:v) in a water bath at 60 °C, and precipitated again by the addition of 305 mL of absolute ethanol for 24 h at 4 °C. The material was centrifuged again and washed successively with 500 mL of absolute ethanol and 300 mL of 80% ethanol. The SPs were dried in an oven at 60 °C for 24 h to obtain the SP-rich fraction. The crude extracts were purified on a DEAE-cellulose resin equilibrated with 0.1 M sodium acetate, 5 mM cysteine, and 5 mM EDTA, pH 5.0. The polysaccharides adsorbed onto the ion exchange resin were eluted with 1.5 M NaCl using gel equilibration buffer. The quantification of SP was carried out through a metachromatic reaction using a spectrophotometer at λ_max_ 525 nm. The high-performance liquid chromatography instrumentation was from Jasco (Jasco Analytical Instruments; 28600 Mary’s Court Easton, MD 21601, USA): Model PU-2080 pump; Chronav system controller; Rheodyne injector equipped with a 20-μL loop; FP-2020 fluorescence detector, MD-2015/2018 diode array arrangement detector and an ELC-2041 evaporative light scattering detector. The TSK G3000SWXL SEC column (0.7 × 300 mm) was equilibrated with phosphate buffer (0.05 M, pH 7.5) for 30 min at a flow rate of 1 mL/min before sample addition. Aliquots of 200 μL of sulfated polysaccharide were loaded on the column. The elution was performed at a flow rate of 1 mL/min and was monitored by UV-Vis detection at λ_max_ 280 nm. For this study, we used the protein markers blue dextran (BD, 2000 kDa); β-amylase (BA, 225 kDa); bovine serum albumin (BSA, Sigma, 66 kDa), which forms a natural dimer of 120 kDa, ovalbumin (OVA, 43 kDa); carbonic anhydrase (CA, 29 kDa); and ribonuclease (RA, 14 kDa) to estimate the molecular weight of the various SP. All samples were analyzed under the same buffer, flow rate and column chromatographic conditions.

### 3.3. Determination of Anti-Leishmanial Activity

*L. (L.) amazonensis* (MHOM/BR/73/M2269 strain) parasite was classified on the basis of characteristic monoclonal antibodies and isoenzymes at the Evandro Chagas Institute, Belém, PA, Brazil. This strain was maintained in the footpad of BALB/c mice to keep their infectivity. The parasites were isolated, cultured, and maintained at 25 °C in RPMI 1640 medium supplemented with 10% heat-inactivated fetal bovine serum, 0.25 mM HEPES, gentamicin (10 μg/mL), and penicillin (100 IU/mL) (R10 medium). The anti-leishmanial assays were performed following the protocol described by Passero *et al*. [13]. Briefly, promastigotes in the stationary phase of growth were washed three times with phosphate buffer solution (PBS) and suspended in RPMI 1640 at a concentration of 107 promastigote/mL in a 96-well plate. The SP were solubilized in PBS and sterilized by filtration through a 0.22 μm membrane before being added to the culture. The SPs were individually added to the culture of *L. (L.) amazonensis* in a range of 20 to 160 μg/mL. After 24 h of incubation at 25 °C, the viability of the promastigote forms were analyzed through the conversion of 3-(4,5-dimethylthiazol)-2,5-diphenyl tetrazolium bromide (MTT) to formazan. Briefly, the plates were washed three times while shaking at 3000 rpm for 10 min at 4 °C, and 40 μL of R10 medium plus 10 μL of MTT (5 mg/mL) were added to each well. After 4 h, 50 μL of 10% sodium dodecyl sulfate was added to each well. The plates were further incubated for 18 h. Next, the plates were read in an ELISA reader at λ_max_ 595 nm, and the 50% effective concentration (IC_50_) of each SP was calculated with the aid of Origin 5.0 statistical software (Microcal Software, Northampton, MA, USA). Each assay was conducted in triplicate. Amphotericin B was used as the standard drug.

### 3.4. Macrophage Cytotoxicity

The cytotoxicity of the SPs was assayed against the mouse macrophage cell line J774 and BALB/c-peritoneal macrophages [14] using a colorimetric reaction based on the MTT assay. Briefly, both cell populations were plated at 106 cell/mL in a 96-well plate. The SPs (at the concentrations described above) were incubated with the cells for 24 h at 35 °C and 5% CO_2_. Following the incubation, the plates were washed three times with PBS, and MTT solution was added as described in the determination of anti-leishmanial activity section. The cytotoxic concentration 50% inhibitory concentration (cytotoxic concentration; CC_50_) of each SP was calculated with the aid of Origin 5.0 statistical software (Microcal Software, Northampton, MA, USA). Each assay was conducted in triplicate.

### 3.5. Selectivity Index Determination

To determine the selectivity index (SI), the ratio of the CC_50_ value of the cytotoxic activity to the EC_50_ value of the antiprotozoal activity was calculated. When the SI value is >10, that compound presents antiprotozoal activity that is higher rather than its cytotoxicity [15].

### 3.6. Lethality Assays

Each SP was separately injected into the tail vein of ten mice in serial doses diluted in 100 μL saline (0.3–40 μg/100 g of body weight). The survival times of the animals were recorded for 24 h.

### 3.7. Statistical Analysis

The results are expressed as the means ± standard deviation. The data were analyzed using Student’s *t* test. The level of significance was set at *p* < 0.05.

## 4. Conclusions

Research with sulfated polysaccharides is one approach for the discovery and development of new drugs that could be used as an alternative to heparin. In the introduction, we presented a brief review of how the heparin-binding proteins (HBPS) that are present on the surface of *Leishmania* spp. may play important roles in the life cycle of parasites and in defining the success of parasite attachment to and invasion of tissues of the mammalian and invertebrate hosts. The relevance of this work is based on the fact that anti-leishmania therapy is based on pentavalent antimony compounds. The toxicity and persistent side effects of these agents are severe, even after modification of the dose level and the duration of treatment [16]. Our results show that the sulfated polysaccharides of algae can be an alternative to the use of heparin in the treatment of leishmaniasis, as these two compounds have similar molecular structures. In addition, the sulfated polysaccharides of algae showed a higher efficiency against the development of leishmaniasis than alkaloids, terpenoids and other natural products isolated from seaweed.

## References

[1] Azevedo-Pereira R.L., Pereira M.C., Oliveira-Junior F.O., Brazil R.P., Cortes L.M., Santos A.L., Toma L., Alves C.R. (2007). Heparin binding proteins from *Leishmania (V.) brasiliensis* promastigotes. Vet. Parasitol..

[2] De Castro Côrtes L.M., de Souza Pereira M.C., da Silva F.S., Pereira B.A., de Oliveira Junior F.O., de Araújo Soares R.O., Brazil R.P., Toma L., Vicente C.M., Nader H.B. (2012). Participation of heparin binding proteins from the surface of *Leishmania (Viannia) braziliensis* promastigotes in the adhesion of parasites to *Lutzomyia longipalpis* cells (Lulo) *in vitro*. Parasit. Vectors.

[3] De Castro Côrtes L.M., de Souza Pereira M.C., de Oliveira F.O., Corte-Real S., da Silva F.S., Pereira B.A., de Fátima Madeira M., de Moraes M.T., Brazil R.P., Alves C.R. (2012). *Leishmania (Viannia) braziliensis*: Insights on subcellular distribution and biochemical properties of heparin-binding proteins. Parasitology.

[4] Rodrigues J.A.G., Farias W.R.L. (2009). Avaliação comparativa dos polissacarídeos sulfatados extraídos de rodofíceas *Halymenia* spp.: Ferramenta taxonômica para algas?. Rev. Bras. Eng. Pesca.

[5] Mayer A.M., Rodríguez A.D., Berlinck R.G., Fusetani N. (2011). arine pharmacology in 2007–8: Marine compounds with antibacterial, anticoagulant, antifungal, anti-inflammatory, antimalarial, antiprotozoal, antituberculosis, and antiviral activities; Affecting the immune and nervous system, and other miscellaneous mechanisms of action. Comput. Biochem. Physiol. C Toxicol. Pharmacol..

[6] Vonthron-Sénécheau C., Kaiser M., Devambez I., Vastel A., Mussio I., Rusig A.M. (2011). Antiprotozoal activities of organic extracts from French marine seaweeds. Mar. Drugs.

[7] Fouladvand M., Barazesh A., Farokhzad F., Malekizadeh H., Sartavi K. (2011). Evaluation of *in vitro* anti-Leishmanial activity of some brown, green and red algae from the Persian Gulf. Eur. Rev. Med. Pharmacol. Sci..

[8] Süzgeç-Selçuk S., Meriçli A.H., Güven K.C., Kaiser M., Casey R., Hingley-Wilson S., Lalvani A., Tasdemir D. (2011). Evaluation of Turkish seaweeds for antiprotozoal, antimycobacterial and cytotoxic activities. Phytother. Res..

[9] Lakshmi V., Kumar R., Gupta P., Varshney V., Srivastava M.N., Dikshit M., Murthy P.K., Misra-Bhattacharya S. (2004). The antifilarial activity of a marine red alga, *Botryocladia leptopoda*, against experimental infections with animal and human filariae. Parasitol. Res..

[10] Choudhary M.I. (2008). Discovery of leishmanicidal agents from medicinal plants. Pure Appl. Chem..

[11] Sabina H., Aliya R. (2011). Bioactive assessment of selected marine red algae against *Leishmania major* and chemical constituents of *Osmundea pinnatifida*. Pak. J. Bot..

[12] Buchmeier N.A., Heffron F. (1989). Intracellular survival of wild-type *Salmonella typhimurium* and macrophage-sensitive mutants in diverse populations of macrophages. Infect. Immun..

[13] Passero L.F., Tomokane T.Y., Corbett C.E., Laurenti M.D., Toyama M.H. (2007). Comparative studies of the anti-leishmanial activity of three *Crotalus durissus* ssp. venoms. Parasitol. Res..

[14] Valentin E., Lambeau G. (2000). Increasing molecular diversity of secreted phospholipases A(2) and their receptors and binding proteins. Biochim. Biophys. Acta.

[15] Lambeau G., Gelb M.H. (2008). Biochemistry and physiology of mammalian secreted phospholipases A2. Annu. Rev. Biochem..

[16] Fagundes F.H.R., Ricardo A., dos Santos M.L., Diz Filho E.B.S., Oliveira S.C.B., Toyama D.O., Toyama M.H. (2011). A catalytically inactive Lys49 PLA2 isoform from *Bothrops jararacussu* venom that stimulates insulin secretion in pancreatic beta cells. Protein Pept. Lett..

